# Ultrasonographic Diagnosis and Conservative Management of Fecal Impaction

**DOI:** 10.7759/cureus.100552

**Published:** 2026-01-01

**Authors:** Wahei Uemura, Tetsuhito Muranaka, Yutaro Otsuka, Yunosuke Takishin, Yasuyuki Kunieda

**Affiliations:** 1 Department of General Practice, Rishiri Island National Health Insurance Central Hospital, Rishiri-cho, JPN; 2 Department of Internal Medicine, Wakkanai City Hospital, Wakkanai, JPN

**Keywords:** bowel obstruction, conservative management, elderly, fecal impaction, point-of-care ultrasound, rectal ultrasound, ultrasonography (usg), ultrasound, ultrasound-guided

## Abstract

Fecal impaction (FI) is a clinically significant condition, especially in the elderly, and may lead to fatal complications, such as stercoral ulceration and intestinal perforation. A 70-year-old man presented with abdominal bloating and was diagnosed with FI using point-of-care ultrasound (POCUS). He was treated with an ultrasound-guided glycerin enema, and the therapeutic effect was monitored in real-time. The patient's condition improved, and he was discharged without requiring surgical intervention. This case demonstrates that POCUS is a valuable tool not only for diagnosing FI but also for dynamically assessing the effectiveness of conservative treatment and guiding management decisions.

## Introduction

Fecal impaction (FI) is a clinically important condition associated with high morbidity, mortality, and increased healthcare costs, particularly in older adults [1]. Its complications are diverse, and a systematic review has summarized several potentially fatal outcomes, such as stercoral ulceration and intestinal perforation [2]. The mortality rate for stercoral perforation, one of the most severe complications, has been reported to exceed 30% [3]. Another study reported an in-hospital mortality rate of 21.9% in FI patients presenting to the emergency department [4].

In Japan, the issue is also becoming more prominent. While a 2000 nationwide survey reported FI as a rare cause of intestinal obstruction [5], a more recent large-scale study found an in-hospital mortality rate of 8.4% among FI patients [6]. These findings highlight the urgent need for rapid and effective diagnostic and management approaches.

Imaging plays a central role in diagnosing intestinal obstruction. Computed tomography (CT) is widely accepted as the gold standard due to its high diagnostic accuracy, with reported sensitivity and specificity of 93% and 100%, respectively [7,8]. However, CT has notable drawbacks, including radiation exposure, cost, and time consumption. As a result, point-of-care ultrasound (POCUS), which can be performed at the bedside, is gaining attention as a rapid, noninvasive alternative. One study demonstrated that POCUS performed by nonspecialist physicians in emergency departments had superior sensitivity compared to plain radiography for detecting small bowel obstruction [9]. Another prospective study found that POCUS had comparable diagnostic accuracy to CT and significantly reduced the time to diagnosis [10]. As summarized in the Supplementary Table 1 (Appendices), previous literature highlights the clinical positioning of POCUS relative to other modalities [7-10]. While CT remains the gold standard with high specificity (79.0%-100.0%), its sensitivity can fluctuate depending on the severity of the obstruction. Notably, the sensitivity of plain radiography is reported to be as low as 46.2% when including all grades of obstruction, significantly underperforming compared to POCUS (83.0%-91.0%).

While CT remains the gold standard for definitive diagnosis and excluding life-threatening complications, this case highlights how POCUS can be utilized as a dynamic tool for monitoring therapeutic progression and guiding clinical management. Although POCUS is becoming a useful tool for the initial assessment of intestinal obstruction, its use has primarily been limited to diagnosis. Reports on its active use in guiding and monitoring conservative treatment are scarce. In this report, we present a case in which POCUS was used not only for diagnosis but also for guiding conservative management and assessing treatment response in real-time, thereby reducing the physical burden on the patient and ultimately avoiding invasive procedures. The primary learning objective of this case is to demonstrate the clinical utility of POCUS-guided glycerin enema placement, combined with the objective assessment of therapeutic response through real-time monitoring of rectal diameter and changes in internal echo patterns. In the diagnostic pathway for suspected FI, POCUS serves as an efficient bedside tool for identifying fecal loading patterns and colonic dilation, allowing for immediate initiation of conservative management. However, CT remains essential for assessing the overall extent of the impaction and excluding critical alternative pathologies or complications, such as obstructing neoplasms, bowel ischemia, or stercoral colitis. By integrating both modalities, clinicians can safely provide POCUS-guided care once life-threatening conditions have been excluded.

## Case presentation

A 70-year-old man presented to our emergency department via ambulance with a three- to four-day history of abdominal bloating and obstipation. He reported intermittent abdominal pain, which prompted the emergency call. The patient had been receiving elobixibat (10 mg daily) and linaclotide (0.25 mg daily) prescribed by his previous physician for chronic constipation; however, his symptoms persisted. Upon arrival, his blood pressure was markedly elevated (165/116 mmHg), which was considered to be associated with the preceding episodes of abdominal pain, although the pain had temporarily subsided at the time of evaluation.

Past medical history

The patient had a history of chronic constipation diagnosed eight years prior, for which he had a pattern of poor adherence to treatment and interrupted medical visits. His other medical history included postnasal drip syndrome (11 years prior), glaucoma (nine years prior), and *Helicobacter pylori *gastritis (treated two years prior). Notably, he underwent surgical repair for a left patellar fracture 15 months prior to admission. The patient achieved independent ambulation without a cane and had no significant limitations in his activities of daily living, though even a minor decline in overall physical activity following the injury was considered a potential contributing factor to his chronic constipation. He also had a history of COVID-19 infection (11 months prior) and colonic polypectomy (10 months prior). His medications at the time of admission included elobixibat (10 mg daily) and linaclotide (0.5 mg daily), both of which had been prescribed for the past seven months. Other medications included loxoprofen (60 mg daily) and ophthalmic solutions (latanoprost and sodium hyaluronate).

Physical examination and vital signs 

He was afebrile (36.3°C), with a blood pressure of 165/116 mmHg, heart rate of 66 bpm, and oxygen saturation of 98% on room air. Abdominal examination revealed distension without tenderness or peritoneal signs. No fever, peritoneal signs, or overt gastrointestinal bleeding were observed. A digital rectal examination confirmed the presence of hard stool in the rectum, but no obvious mass lesion or blood was palpated.

Investigations

Initial ultrasonography revealed a hyperechoic shadow in the rectum with posterior acoustic shadowing suggestive of hardened stool. The transverse rectal diameter was measured at 46 mm, indicating significant fecal loading. Furthermore, ultrasound demonstrated marked proximal colonic dilatation extending to the ascending colon. While routine rectal constipation typically involves isolated fecal loading in the rectum, this proximal colonic distension indicated an obstructive pattern. This specific linkage between rectal impaction and upstream bowel dilation allowed us to differentiate FI from simple functional constipation and led to the immediate decision. No evidence of tumor or ischemic changes was noted. Abdominal radiographs showed colonic air-fluid levels. A CT scan was subsequently performed to confirm the extent of the impaction and to exclude underlying organic diseases or complications such as stercoral colitis. Based on these findings, a diagnosis of fecal intestinal obstruction was established.

Management and hospital course

The patient was admitted for conservative management. A plan was made for bowel rest with intravenous fluid administration and serial evaluation with POCUS.

Day 1

**Figure 1:**
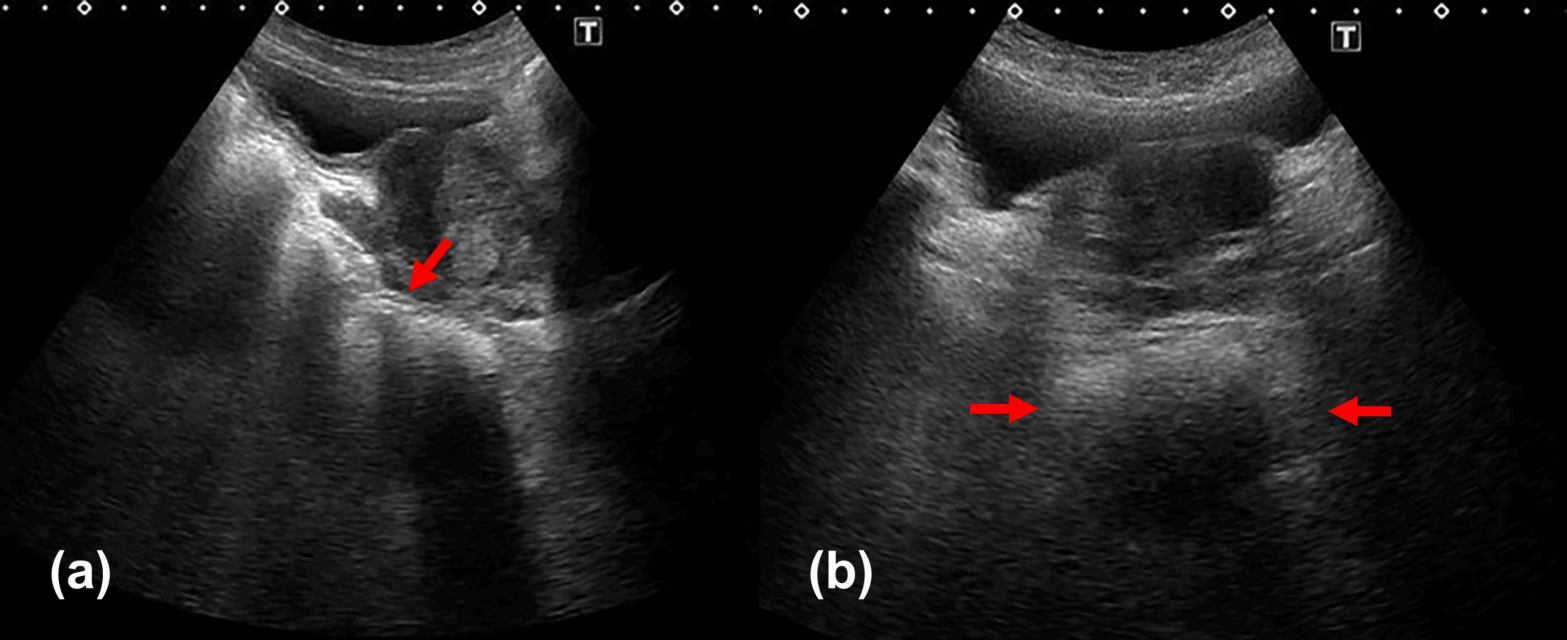
Short-axis and long-axis ultrasonographic images of the rectum on day 1 (a) A hyperechoic mass with posterior acoustic shadowing is noted in the rectum, suggestive of a fecal impaction. The rectum and proximal colon are dilated and filled with hyperechoic material, consistent with constipation. (b)The transverse diameter of the rectal distension on the short-axis view measures approximately 46 mm. Rectal diameter was measured from outer-wall to outer-wall in the transverse plane, following the method described by Yabunaka et al. [11]. The patient was examined in the supine position. Images were obtained using an Aplio 500 ultrasound system (Canon Medical Systems Corporation, Otawara, Japan) with a convex probe set at 3.5 MHz

**Figure 2:**
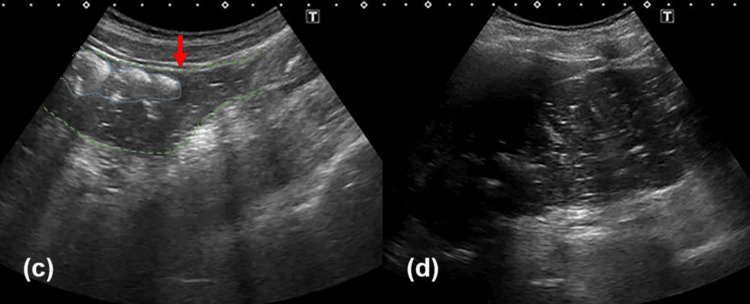
Ultrasound demonstrated colonic dilatation involving both the ascending and descending colon on day 1 (c) Ultrasound demonstrated colonic dilatation involving the sigmoid colon. The colon is outlined by the green dashed line, and the blue dashed line indicates hard stool characterized by a hyperechoic surface image. A transition from solid to liquid stool was evident at the sigmoid colon (indicated by the red arrow), supporting the diagnosis of a distal colonic obstruction. (d) Ultrasound demonstrated colonic dilatation involving the ascending colon. Intraluminal anechoic fluid collections with punctate echogenic foci were observed, consistent with liquid stool. The patient was examined in the supine position. Images were obtained using an Aplio 500 ultrasound system (Canon Medical Systems Corporation, Otawara, Japan) with a convex probe set at 3.5 MHz

Ultrasound confirmed the diagnosis of fecal intestinal obstruction, revealing a large fecal mass with posterior acoustic shadowing (Figure 1a) and a rectal diameter of 46 mm (Figure 1b). The obstruction was further characterized by colonic dilation with a discernible transition point from solid to liquid stool in the sigmoid colon (Figure 2c), extending proximally to the ascending colon (Figure 2d). The patient was placed on nil per os (NPO) status and received intravenous fluids.

Day 2

As acute-phase treatment, an ultrasound-guided glycerin enema (120 mL, 50% solution) was performed. POCUS was used to verify proper catheter placement in the rectum before instillation, minimizing the risk of mucosal injury. After administration, an anechoic fluid collection was observed around the fecal mass (Video 1). POCUS confirmed fragmentation of the fecal mass and resolution of the obstruction. Therefore, oral polyethylene glycol (PEG) was initiated as maintenance therapy.

**Video 1:** Bedside ultrasound-guided glycerin enema administration using a pocket-sized ultrasound POCUS: point-of-care ultrasound (g) Real-time POCUS visualizes the anterior-posterior movement of the enema catheter tip (hyperechoic linear artifact) and the instillation of the enema solution, ensuring correct positioning within the rectum to avoid mucosal injury. (h) Post-enema image demonstrating the hyperechoic hard stool within the rectum surrounded by an anechoic fluid collection (instilled enema solution). The patient was examined in the supine position. The procedure was performed using 120 mL of a 50% glycerin solution and a wireless handheld ultrasound device (Vscan Air CL, GE HealthCare, Chicago, IL, USA) with a curved array transducer (2-5 MHz)

Day 3

Although abdominal bloating persisted, the patient's vital signs remained stable, and bowel rest was continued.

Day 4

As spontaneous defecation had not occurred, a re-evaluation with POCUS was performed. It revealed re-accumulation of stool and distention in the rectum; therefore, a second ultrasound-guided glycerin enema (120 mL, 50% solution) was administered.

Day 5

**Figure 3:**
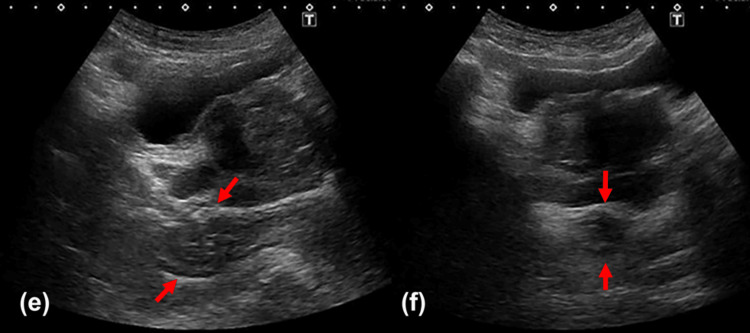
Short-axis and long-axis ultrasonographic images of the rectum on day 5 (e) In the long-axis view, the posterior wall of the rectum was visualized. Due to bowel collapse, the layered structure of the rectal wall was prominent (indicated by the red arrow). (f) In the short-axis view, the posterior wall of the rectum was also visualized (indicated by the red arrow). Compared to day 1, the rectal distension measured approximately 22 mm, indicating a decrease in luminal diameter. The patient was examined in the supine position. Images were obtained using an Aplio 500 ultrasound system (Canon Medical Systems Corporation, Otawara, Japan) with a convex probe set at 3.5 MHz

POCUS showed the posterior wall of the rectum (Figure 3e) and a reduction in the rectal diameter to 22 mm (Figure 3f). The patient began passing soft stools on the same day.

Day 6

Oral intake was well-tolerated, and with no recurrence of symptoms, the patient was discharged.

Follow-up

During outpatient follow-up, POCUS examinations were performed at one and three months postdischarge to ensure no recurrence of fecal impaction and to evaluate the therapeutic effectiveness of PEG, which was initiated as maintenance therapy for chronic constipation. These examinations confirmed no recurrence of FI and showed well-controlled fecal loading in the rectum. Additionally, a colonoscopy was performed two months postdischarge to rule out organic diseases, such as obstructing neoplasms. It revealed a 6 mm sigmoid colon polyp, which was determined to be unrelated to the episode of FI and was removed via endoscopic mucosal resection without complications.

## Discussion

The most important clinical insight from this case is that POCUS can be used not only as a diagnostic tool for FI but also as a practical method to guide therapeutic interventions in real time and to objectively assess treatment effectiveness.

In this case, transabdominal ultrasound was used to quantitatively measure rectal diameter, offering an objective and reproducible diagnostic approach. This method has been validated in pediatric populations, with a recent meta-analysis confirming its utility in diagnosing functional constipation and FI in children [11]. Expert consensus suggests that a rectal diameter of ≥30 mm indicates FI in pediatric patients [12]. In our case, the rectal diameter was 46 mm, which clearly exceeded this threshold and was consistent with previously reported values, such as an average rectal diameter of 42.1 mm in children with constipation [13]. Although validated adult diagnostic criteria are still lacking, a preliminary study by Yabunaka et al. on healthy adults reported a mean rectal diameter of 4.22 ± 0.8 cm when a defecation desire was present. Furthermore, they found that hard stools (Bristol Stool Scale 1 or 2) were associated with high echo areas and acoustic shadows in 100% of cases. Our finding of a 46 mm diameter on day 1 is consistent with these reports, suggesting that this quantitative measure can serve as an objective indicator of significant fecal loading and treatment response in adults [14].

The key contribution of this report, however, lies in extending the role of POCUS beyond diagnosis. In pediatrics, rectal diameter has been used to guide decisions regarding disimpaction, with 3 cm often cited as a reference point [15]. In this case, ultrasound was not only used to confirm the diagnosis but also to guide the safe placement of the enema catheter and to monitor the reduction in fecal burden after treatment. This real-time feedback provided objective evidence that conservative treatment was effective and helped avoid unnecessary surgical intervention.

This approach aligns with broader clinical trends in ultrasound-guided minimally invasive therapies for obstructive conditions. For instance, Kang et al. reported successful use of ultrasound-guided endoscopic retrograde appendicitis therapy (ERAT) in children with chronic appendicitis due to fecaliths, thereby avoiding surgery [16]. This supports the fundamental concept of ultrasound serving as a therapeutic as well as a diagnostic tool.

The clinical implications of this strategy are substantial. Particularly in resource-limited settings such as remote islands, rural clinics, or home care environments, POCUS enables clinicians to complete the diagnostic and treatment process at the bedside. This may reduce radiation exposure, avoid invasive procedures, and enhance patient safety and comfort. For older adults, in whom FI significantly impairs quality of life [17], this minimally invasive approach can also reduce physical burden and improve care outcomes. The small sigmoid colon polyp discovered during follow-up colonoscopy was deemed incidental and unrelated to the FI episode.

This report has several limitations. First, as a single-case design, the findings regarding management effectiveness cannot be generalized; further large-scale studies are required to confirm the safety and efficacy of POCUS-guided management. Second, POCUS is inherently operator-dependent, meaning that the accuracy of rectal diameter measurements and the success of procedural guidance rely significantly on the clinician's skill and experience. Third, while we referenced preliminary adult data [14], universally validated diagnostic thresholds for rectal diameter in elderly populations are still lacking. Finally, although CT was utilized in this case to ensure diagnostic accuracy and exclude complications, the feasibility of a POCUS-only approach in various clinical settings remains a subject for future research.

## Conclusions

This case demonstrates the feasibility and potential utility of POCUS as a versatile dynamic monitoring tool for managing fecal impaction, extending its role beyond initial diagnosis to real-time procedural guidance. While our findings suggest a promising, noninvasive alternative for reducing clinical burden, they are limited by a single-case design and do not establish comparative effectiveness against usual care. Future research is warranted to establish standardized adult protocols and validate the broader clinical impact through larger-scale studies.

## Appendices

**Table 1:** Diagnostic accuracy of various imaging modalities for intestinal obstruction POCUS: point-of-care ultrasound This table summarizes the sensitivity and specificity of major imaging modalities for the diagnosis of intestinal obstruction based on key prospective and retrospective studies. While CT remains the gold standard for definitive diagnosis, POCUS demonstrates comparable sensitivity, significantly outperforming plain radiography. These data support the use of POCUS as a reliable, rapid bedside tool for initial triage and monitoring in clinical practice. Note: Values represent ranges reported across key prospective and retrospective studies. *For CT and Plain X-ray, the higher end of the sensitivity range reflects performance in high-grade obstruction, while the lower end includes studies with lower-grade obstruction

Modality	Sensitivity	Specificity	References
POCUS	83.0%-91.0%	75.3%-100.0%	[8-10]
CT scan	81.0%-94.0%*	79.0%-100.0%	[7,8]
Plain X-ray	46.2%-86.0%*	57.0%-85.0%	[7-9]

## References

[1] Hussain ZH, Whitehead DA, Lacy BE (2014). Fecal impaction. Curr Gastroenterol Rep.

[2] Serrano Falcón B, Barceló López M, Mateos Muñoz B, Álvarez Sánchez A, Rey E (2016). Fecal impaction: a systematic review of its medical complications. BMC Geriatr.

[3] Chakravartty S, Chang A, Nunoo-Mensah J (2013). A systematic review of stercoral perforation. Colorectal Dis.

[4] Sommers T, Petersen T, Singh P (2019). Significant morbidity and mortality associated with fecal impaction in patients who present to the emergency department. Dig Dis Sci.

[5] Onda M, Takasaki H, Furukawa K, Tanaka N, Moriyama Y (2000). Nationwide investigation of 21,899 cases of intestinal obstruction. J Abdom Emerg Med.

[6] Hoshi H, Endo A, Fushimi K, Morishita K (2025). Socioeconomic burden of patients hospitalized for fecal impaction: a nationwide retrospective observational study. BMC Gastroenterol.

[7] Burkill G, Bell J, Healy J (2001). Small bowel obstruction: the role of computed tomography in its diagnosis and management with reference to other imaging modalities. Eur Radiol.

[8] Suri S, Gupta S, Sudhakar PJ, Venkataramu NK, Sood B, Wig JD (1999). Comparative evaluation of plain films, ultrasound and CT in the diagnosis of intestinal obstruction. Acta Radiol.

[9] Jang TB, Schindler D, Kaji AH (2011). Bedside ultrasonography for the detection of small bowel obstruction in the emergency department. Emerg Med J.

[10] Boniface KS, King JB, LeSaux MA, Haciski SC, Shokoohi H (2020). Diagnostic accuracy and time-saving effects of point-of-care ultrasonography in patients with small bowel obstruction: a prospective study. Ann Emerg Med.

[11] Yabunaka K, Matsumoto M, Yoshida M (2018). Assessment of rectal feces storage condition by a point-of-care pocket-size ultrasound device for healthy adult subjects: a preliminary study. Drug Discov Ther.

[12] Vos JM, Bloem MN, de Geus A (2024). Accuracy of transabdominal ultrasound to diagnose functional constipation and fecal impaction in children: a systematic review and meta-analysis. Pediatr Radiol.

[13] Mathias RM, Goodsall TM, Parker CE (2025). Expert position statement: defining the role of intestinal ultrasound in assessing constipation and faecal loading. Aliment Pharmacol Ther.

[14] Joensson IM, Siggaard C, Rittig S, Hagstroem S, Djurhuus JC (2008). Transabdominal ultrasound of rectum as a diagnostic tool in childhood constipation. J Urol.

[15] Di Pace MR, Catalano P, Caruso AM, Bommarito D, Casuccio A, Cimador M, De Grazia E (2010). Is rectal disimpact always necessary in children with chronic constipation? Evaluation with pelvic ultrasound. Pediatr Surg Int.

[16] Kang JQ, Zhang W, Zhang YL (2022). Application of ultrasound-guided endoscopic retrograde appendicitis therapy in children with appendix-related chronic abdominal pain (Article in Chinese). Zhongguo Dang Dai Er Ke Za Zhi.

[17] De Lillo AR, Rose S (2000). Functional bowel disorders in the geriatric patient: constipation, fecal impaction, and fecal incontinence. Am J Gastroenterol.

